# The Effectiveness of Three Regions in Mitochondrial Genome for Aphid DNA Barcoding: A Case in Lachininae

**DOI:** 10.1371/journal.pone.0046190

**Published:** 2012-10-03

**Authors:** Rui Chen, Li-Yun Jiang, Ge-Xia Qiao

**Affiliations:** 1 Key Laboratory of Zoological Systematics and Evolution, Institute of Zoology, Chinese Academy of Sciences, Chaoyang District, Beijing, People's Republic of China; 2 Graduate University of Chinese Academy of Sciences, Shijingshan District, Beijing, People's Republic of China; Université Joseph Fourier, France

## Abstract

**Background:**

The mitochondrial gene *COI* has been widely used by taxonomists as a standard DNA barcode sequence for the identification of many animal species. However, the *COI* region is of limited use for identifying certain species and is not efficiently amplified by PCR in all animal taxa. To evaluate the utility of *COI* as a DNA barcode and to identify other barcode genes, we chose the aphid subfamily Lachninae (Hemiptera: Aphididae) as the focus of our study. We compared the results obtained using *COI* with two other mitochondrial genes, *COII* and *Cytb*. In addition, we propose a new method to improve the efficiency of species identification using DNA barcoding.

**Methodology/Principal Findings:**

Three mitochondrial genes (*COI*, *COII* and *Cytb*) were sequenced and were used in the identification of over 80 species of Lachninae. The *COI* and *COII* genes demonstrated a greater PCR amplification efficiency than *Cytb*. Species identification using *COII* sequences had a higher frequency of success (96.9% in “best match” and 90.8% in “best close match”) and yielded lower intra- and higher interspecific genetic divergence values than the other two markers. The use of “tag barcodes” is a new approach that involves attaching a species-specific tag to the standard DNA barcode. With this method, the “barcoding overlap” can be nearly eliminated. As a result, we were able to increase the identification success rate from 83.9% to 95.2% by using *COI* and the “best close match” technique.

**Conclusions/Significance:**

A *COII*-based identification system should be more effective in identifying lachnine species than *COI* or *Cytb*. However, the *Cytb* gene is an effective marker for the study of aphid population genetics due to its high sequence diversity. Furthermore, the use of “tag barcodes” can improve the accuracy of DNA barcoding identification by reducing or removing the overlap between intra- and inter-specific genetic divergence values.

## Introduction

DNA sequences can be useful tools for the taxonomic identification of biological materials [1]. DNA barcoding has been proposed as a method to efficiently describe biodiversity. The mitochondrial gene encoding cytochrome c oxidase I (*COI*) has been widely used as a DNA barcode in many animal species [2]. However, numerous problems have arisen with the use of this gene as a barcode [3]–[6], largely due to its low level of variation among species [7]–[11]. These issues suggest that DNA barcode technology should be improved and that DNA barcode genes should be identified that more efficiently distinguish among species.

Aphids are a group of phloem-feeding insects belonging to the order Hemiptera, which comprises more than 4700 species [12]–[13]. Aphids of the subfamily Lachninae [14] feed on plants in Coniferae, on some broad-leaf plants and on the roots of some weed species [15]. There are 339 known species of Lachninae distributed among 18 genera, composing 3 tribes worldwide. Lachninae are an ideal target for DNA barcoding as some genera in the subfamily feed on specific host plants, which facilitates identification at the genus level by morphological characteristics and unique host plants. However, the identification of congeneric Lachninae species is difficult due to the shortage of effective morphological characteristics available to distinguish among the large number of closely related species.

To evaluate the effectiveness of alternative mitochondrial genes as DNA barcodes and to identify useful markers that will improve the rate of species identification, we chose to focus on Lachninae as a model system. We examined three mitochondrial genes as molecular markers, *COI* (657 bp), *COII* (671 bp) and *Cytb* (730 bp), and we compared the utility of these genes in demonstrating sequence diversity sufficient to efficiently distinguish among species. We sequenced and analyzed 1098 sequences from 3 mitochondrial genes representing 83 species in 14 genera and the 3 tribes of Lachninae. In addition, we propose a new method to improve the DNA barcoding identification system using the optimal markers.

## Results

### Saturation analysis

We tested the substitution saturation in 3 mitochondrial gene sequences using DAMBE 4.5.20. The results revealed that the sequence transitions and transversions in each gene demonstrated a linear relationship (Figure S1), thus permitting their use in DNA barcoding.

### Efficiency of PCR amplification

We calculated the efficiency of PCR amplification of *COI*, *COII* and *Cytb* sequences for all samples and determined that the rate of success was 95.51%, 99.44% and 82.58%, respectively, with *COII* demonstrating the highest probability of amplification. The *COI* primers had a 2.92% probability of amplifying sequences from parasitic wasps, which frequently lay their eggs in aphids. Primers for *COII* and *Cytb* genes did not show nonspecific amplification.

### NJ Tree structure

**Figure 1:**
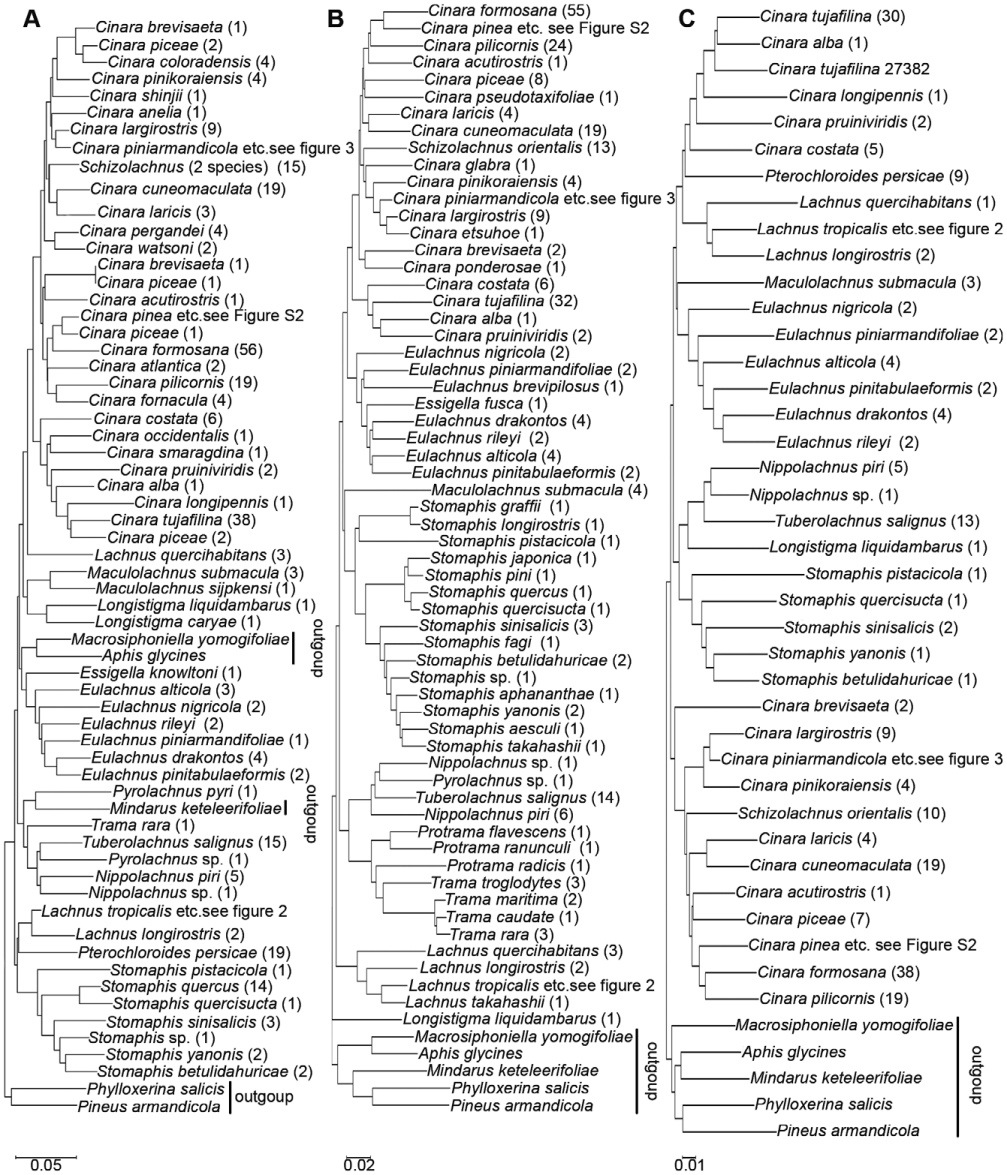
Basal nodes of the NJ tree based on distances obtained using the K2P model. A, B, and C are the *COI*, *COII* and *Cytb* NJ trees, respectively. The number of identical samples is given in brackets.

The results of the overall NJ analysis of the *COI*, *COII* and *Cytb* regions of distances among samples representing 83 species are summarized in Figure 1. It should be noted that the trees presented here are intended to represent only the distance matrix and should not be interpreted as a phylogenetic hypothesis.

**Figure 2:**
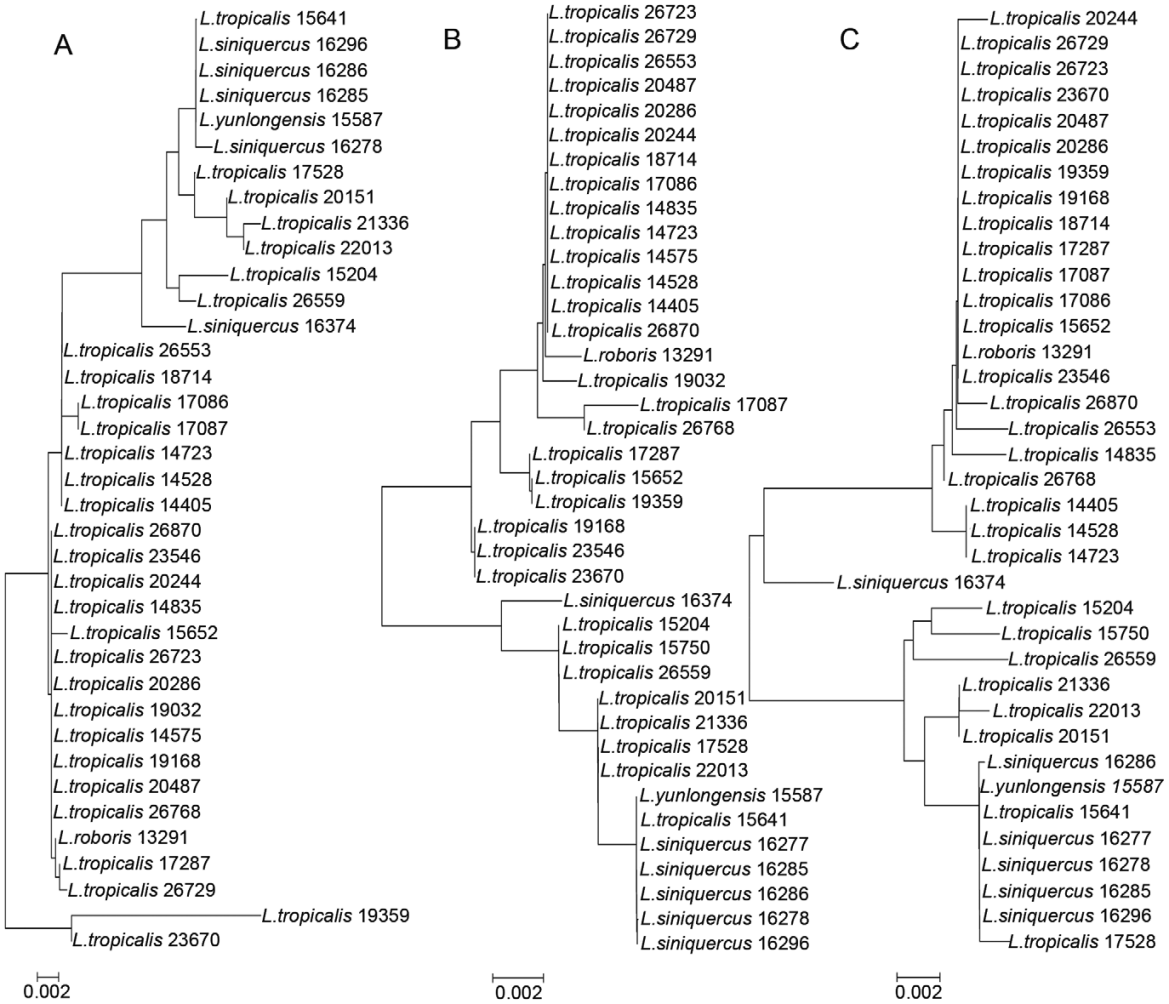
NJ analysis of K2P distances in *Lachnus siniquercus* Zhang, *L. tropicalis* (van der Goot), *L. roboris* (Linnaeus) and *L. yunlongensis* Zhang. A, B, and C are the *COI*, *COII* and *Cytb* NJ trees, respectively.

In the three NJ trees, the majority of species formed distinct clusters, except for some species such as *L. siniquercus*, *L. tropicalis*, *L. roboris* and *L. yunlongensis* in the genus *Lachnus* (Figures 1, 2) and *C. pinea*, *C. atrotibialis* and *C. piniphila* in the genus *Cinara* (Figures 1, S2). Species in outgroups were embedded in Lachninae in the *COI* tree, while in the *COII* and *Cytb* trees, the ingroups formed a monophyletic group within Lachninae. In the *COII* and *Cytb* trees, both *C. piceae* and *C. brevisaeta* clustered together, respectively; but in the *COI* tree, both species did not form their own cluster, respectively. Due to its high intraspecific divergence, *C. piniarmandicola* formed a polyphyletic group in the *Cytb* tree. A greater number of species formed single clusters in the *COII* tree compared to the *COI* and *Cytb* trees. In addition, we amplified 10 sequences from parasitic wasps using primers for the *COI* region, and these non-specific sequences did not appear in the *COII* and *Cytb* trees.

### Data analysis

For the *COI* sequence, 658 bp was used for data analysis, among which 345 sites were conserved, 313 were variable and 264 were parsimony-informative. These sequences are heavily biased toward A and T nucleotides, averaging 40.1% T, 14.2% C, 35.5% A and 10.2% G. For the *COII* sequence, 671 bp was used for data analysis. Of these nucleotides, 335 sites were conserved, 333 were variable and 295 were parsimony-informative. These sequences are similarly heavily biased toward A and T nucleotides, averaging 40.1% T, 12.5% C, 40.0% A and 7.4% G. For the *Cytb* sequence, 730 bp was used for data analysis. Of these nucleotides, 350 sites were conserved, 380 were variable and 327 were parsimony-informative. The average nucleotide composition for *Cytb* is 42.4% T, 12.9% C, 35.8% A and 9.0% G.

The interspecific divergence of congeneric species was assessed by average inter-specific distance (K2P distance) among the species within a genus, each of which was represented by at least two species in this study (Table 1). The intra-specific divergence of congeneric species was evaluated by 3 additional parameters: the average intra-specific difference, theta (θ) and average coalescent depth [4], [16]. The average intra-specific difference (K2P distance) was determined for all samples collected within each species that was represented by more than one individual. The theta (θ) value was calculated as the mean pairwise distance within each species with at least two representatives, which eliminates the bias associated with unequal sampling among individuals in a species. The average coalescent depth was determined to be the maximum intra-specific distance within each species that was represented by at least two individuals (Table 1).

**Table 1:** Analysis of intra- and interspecific divergence among congeneric species in selected genera of Lachninae.

	Analysis of intra- and inter-specific divergences of congeneric species in Lachninae			
Genera	Average inter-specific distance (*COI*/*COII*/*Cytb*)	Average intra-specific distance (*COI*/*COII*/*Cytb*)	Theta (θ) (*COI*/*COII*/*Cytb*)	Coalescent depth (*COI*/*COII*/*Cytb*)
*Cinara* Curtis	0.0875±0.0222	0.0198±0.0185	0.0225±0.0262	0.0499±0.0414
	0.0981±0.0222	0.0196±0.0202	0.0157±0.0133	0.0332±0.0296
	0.1039±0.0237	0.0207±0.0224	0.0162±0.0117	0.0388±0.0320
*Eulachnus* del Guercio	0.0772±0.0090	0.0074±0.0113	0.0108±0.0162	0.0125±0.0161
	0.0781±0.0130	0.0064±0.0098	0.0078±0.0141	0.0087±0.0143
	0.0954±0.0144	0.0083±0.0133	0.0110±0.0197	0.0123±0.0199
*Lachnus* Burmeister	0.0436±0.0369	0.0094±0.0095	0.0064±0.0003	0.0202±0.0198
	0.0443±0.0370	0.0065±0.0062	0.0045±0.0028	0.0099±0.0083
	0.0568±0.0521	0.0098±0.0095	0.0069±0.0055	0.0129±0.0099
*Stomaphis* Walker	0.0867±0.0221	0.0031±0.0042	0.0017±0.0003	0.0026±0.0045
	0.0820±0.0272	0.0043±0.0058	0.0024±0.0041	0.0036±0.0062
	0.1192±0.0250	0.0125±0.0000	0.0125±0.0000	0.0125±0.0000

The inter-specific divergence of con-tribe species was assessed by the average inter-specific distance (K2P distance) between all species within the genera of each tribe that were represented by at least two species (Table 2). The mean intra-specific nucleotide divergence was determined for all of the samples collected within a species that was represented by more than one individual (Table S3).

**Table 2:** Analysis of interspecific divergence of species within the tribes of Lachninae.

	Analysis inter-specific divergences of species within con-tribe in Lachninae		
Tribes	*COI*	*COII*	*Cytb*
Cinarini	0.1039±0.0188	0.1073±0.0137	0.1142±0.0127
Lachnini	0.1272±0.0179	0.1274±0.0209	0.1395±0.0225

### Success of similarity-based DNA identification techniques

The rate of success using the “best match” technique was 92.7% for *COI*, 96.9% for *COII* and 93.8% for *Cytb*. There were 11 (2.7%), 12 (3.1%) and 19 (6.3%) sequences of *COI*, *COII* and *Cytb*, respectively, that yielded ambiguous results. A total of 19 *COI* sequences were misidentified (Table 3). The data set contained 1098 sequences, including 409 *COI* sequences, 385 *COII* sequences and 304 *Cytb* sequences. The best match for each sequence was an identical match.

**Table 3:** Successful identification of species based on “best match” and “best close match” analysis of the interspecific divergence of *COI*, *COII* and *Cytb* sequences among species within Lachninae.

Parameter	“Best match”	“Best close match”
Success	92.7%/96.9%/93.7%	83.9%/90.8%/83.7%
Ambiguous	2.7%/3.1%/6.3%	11.0%/8.0%/14.5%
Misidentification	4.6%/0.0%/0.0%	5.1%/1.2%/1.8%

To use the “best close match” technique, we determined that 95% of the intra-specific distances for the 3 markers fell between 0% and 5.54%, and the latter value was used to decide whether a query had a close enough barcode match for identification. The rate of success using this method was 83.9%, 90.8% and 83.7% for *COI*, *COII* and *Cytb*, respectively (Table 3).

## Discussion

The mitochondrial gene *COI* has been routinely used for species identification across the animal kingdom [2]. However, the interspecsific sequence divergence of *COI* and the divergence among congeneric species are highly variable depending on the different animal groups. In addition, *COI* is used to study lower-level relationships, such as those among species within a genus [17]–[18]. Despite its widespread use, however, the following question remains: is *COI* the most suitable DNA barcode for aphid identification?

### DNA barcodes may fail to identify morphologically similar species

In our analysis, we observed that in some cases, the genetic divergence between congeneric species approached zero. These results indicated that some species groups could not be distinguished by mitochondrial gene sequences. The following analysis examines the relationships between *Lachnus* species with a genetic divergence of less than 1%.

*Lachnus siniquercus* Zhang, *L. tropicalis* (van der Goot), *L. roboris* (Linnaeus) and *L. yunlongensis* Zhang are four related species of the genus *Lachnus*. These four species clustered together in the 3 NJ trees (Figure 2).

All four of these species feed on plants of the Fagaceae family and *L. yunlongensis* Zhang also feeds on willow. The average divergence among the species at the three markers was 0.8% for *COI* (ranging from 0.5 to 2.5%), 0.9% for *COII* (ranging from 0.0 to 1.8%) and 1.0% for *Cytb* (ranging from 0.0 to 2.1%). According to our criteria, therefore, these four species are indistinguishable by the three DNA barcoding markers.

The morphological characteristics of the four species are also similar (Table 4). Differential characteristics display nearly continuous variation, making identification by morphology similarly problematic.

**Table 4:** Morphological characteristics of *Lachnus siniquercus* Zhang, *L. tropicalis* (van der Goot), *L. roboris* (Linnaeus) and *L. yunlongensis* Zhang.

	Morphological characteristics					
Species	Body length (mm)	Ratio of antennal (segment IV and III)	The number of setae of abdominal tergite VIII	Ratio of setae length of antennal segment III and widest diameter of the segment	Length ratio of hind tibia and body	The number of setae on cauda
*Lachnus siniquercus*	5.1	0.50	37–55	0.50	0.73	60
*Lachnus tropicalis*	3.1	0.33	18–25	1.10	0.97	24–35
*Lachnus roboris*	4.4	0.41	48–49	0.79	0.87	38–55
*Lachnus yunlongensis*	4.5	0.45	43–44	0.67	0.82–0.87	62–75

A similar observation can be made for other Lachninae genera that are difficult to identify based on morphological characteristics. In the genus *Cinara*, for example, three species, *C. pinea* (Zetterstedt), *C. atrotibialis* David & Rajasingh and *C. piniphila* (Ratzeburg), clustered together in all three NJ trees (Figure S2). Similar difficulties occur in other taxa, such as birds [19], butterflies [20], fish [21] and plants [7]–[9]. As DNA barcodes may fail to identify morphologically similar species, it is important for taxonomists and molecular biologists to consider the best method for classifying these species and to determine if these species are synonymous.

### *Cytb* leads to ambiguous species identification

During our analysis of genetic divergence using *Cytb*, we observed intra-specific genetic divergence values of greater than 6% in some samples, especially in species from geographically distant locations, such as *Cinara tujafilina* (del Guercio), *C. formosana* (Takahashi) and *C. pinea* (Zetterstedt). For example, *C. tujafilina* (del Guercio) is widely distributed throughout China and displays a wide range of intra-specific divergence values among samples from different locations. Geographical populations show a wealth of nucleotide diversity. We observed a mean intra-specific nucleotide divergence of 0.0235 for *COI*, 0.0257 for *COII* and 0.0336 for *Cytb* (Table S3).

**Figure 3:**
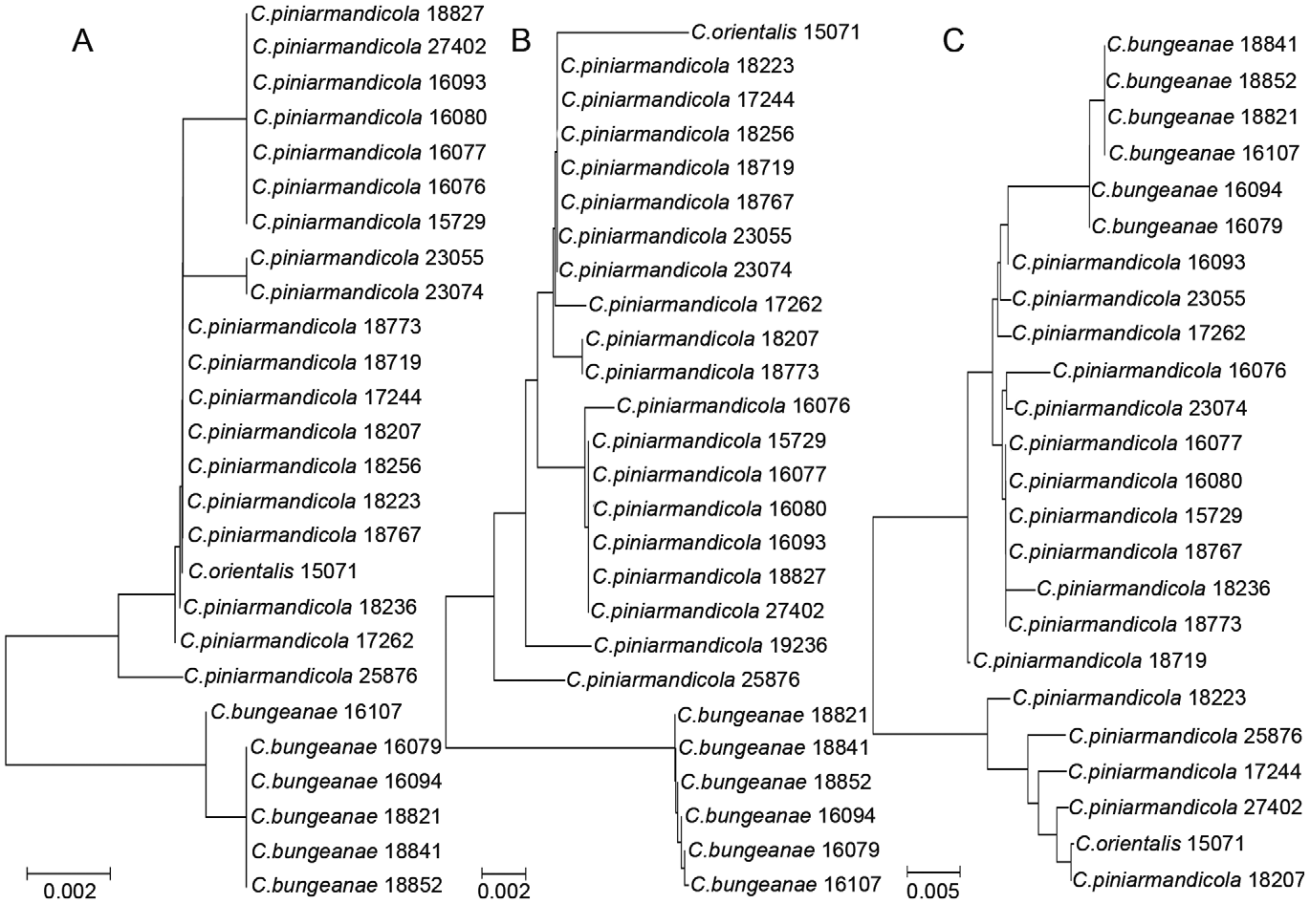
NJ analysis of K2P distances in *Cinara piniarmandicola* Zhang, Zhang & Zhong, *C. bungeanae* Zhang, Zhang & Zhong and *C. orientalis* (Takahashi). A, B, and C are the *COI*, *COII* and *Cytb* NJ trees, respectively.

The *Cytb* sequence appears to be more effective and informative than other genes for the study of aphid population genetics. However, due to a high level of nucleotide diversity, analysis of *Cytb* often led to ambiguous species identification (6.2% in the “best match” analysis and 14.5% in the “best close match” analysis). An example of the utility of *Cytb* occurred in the cluster comprising *Cinara piniarmandicola* Zhang, Zhang *&* Zhong, *C. bungeanae* Zhang, Zhang *&* Zhong and *C. orientalis* (Takahashi). *C. piniarmandicola* and *C. orientalis* cannot be distinguished from each other and were regarded as a whole. Using the *COI* and *COII* sequences, the two species formed a single cluster. In contrast, in the *Cytb* tree, they formed different clusters (Figure 3), as the maximum intra-specific divergence of *C. piniarmandicola* (0.0488) based on *Cytb* gene is higher than the inter-specific divergence between *C. piniarmandicola* and *C. bungeanae* (0.0222, SE = 0.0119).

### Comparison among the three mitochondrial regions

In Lachninae, the absolute amount of intra-specific divergence among *COI* and *Cytb* sequences was generally equal to or greater than that of the *COII* sequence. For example, in *Lachnus*, the average intra-specific divergence of *COI* was 0.0094 (SE = 0.0095) and of *Cytb* was 0.0098 (SE = 0.0095); both were greater than the divergence of *COII* (0.0065, SE = 0.0062). Though for some species the analysis of *Cytb* leads to ambiguous species identification, in other cases, it can provide useful information on population structure or on subtle differences within a species. An example of this can be found in *Cinara tujafilina* (del Guercio), the intra-specific *Cytb* divergence of which can reach 0.0843.

The interspecific divergence of *COI* varies in different animal group. Among the 13,320 species pairs of animals, the divergence ranged from 0.00 to 0.54, while most pairs of species (79%) showed sequence divergence greater than 0.08 [2], and the mean sequence divergence among congeneric species was 0.18 for mayflies [22] and 0.164 for spiders [23]. In Lachninae, the inter-specific divergence of *COI* was always less than the corresponding divergence of *COII* or *Cytb* (Tables 1, 2). For example, in *Cinara*, the average inter-specific divergence of *COI* among congeneric species was 0.0875 (SE = 0.0222), which was lower than the divergence of *COII* (0.0981, SE = 0.0222) and of *Cytb* (0.1039, SE = 0.0237) (Table 1). When we calculated the inter-specific divergence of species within the same tribe, *COI* likewise displayed the lowest divergence of the three markers (Table 2). In addition, Lachninae cannot form a monophyletic group in the *COI* tree, while in the *COII* and *Cytb* trees, the family is monophyletic (Figure 1). Under Hebert's criterion [2], using this sequence to identify a species would fail because the group is not monophyletic. Compared the efficiency of PCR amplification among three markers, COII was best and Cytb was worst. In addition, the *COI* gene yielded greater nonspecific amplification (2.92%) than the other two markers.

Species were identified using the “best match” and “best close match” approaches. Analysis of the *COII* sequence yielded an identification frequency for the “best match” and “best close match” techniques of 96.9% and 90.8%, respectively (Table 3), which was significantly higher than that for the other two markers. The frequency of successful identification using *COI* and *Cytb* was similar for both approaches (92.7% and 93.7% for the “best match” method and 83.9% and 83.7% for the “best close match” method, respectively). Thus, *COII* yielded the most accurate identification of Lachninae species in our study.

Our results suggest that *COII* is a more reliable indicator of intra- and interspecies divergence than *COI* or *Cytb*. In addition, *COII* was efficiently amplified by PCR and provided accurate species identification. As species identification problems arose mainly in the case of indistinguishable sequences between species, a *COII*-based identification system should be effective in distinguishing Lachninae species from one another. However, at the level of both genus and tribe, there was significant overlap in all three markers. Similarly, none of the markers could easily distinguish morphologically similar species from each other (Figures 2, S2).

### A novel method to improve the DNA barcoding identification system

The use of DNA barcoding as a method for rapid species identification and for the discovery of new groups requires that inter-specific variation exceeds intra-specific variation by one order of magnitude to establish a “barcoding gap” or the reciprocal monophyly of a species. In many cases, however, a “barcoding gap” is absent [24].

In our study, the “barcoding gap” was not present, and significant overlaps were formed in Lachninae using the *COI*, *COII* and *Cytb* markers. This overlap resulted in a high probability of ambiguous species identification or of species misidentification (Table 3). Therefore, we propose a new method to improve molecular species identification called “tag barcodes.” In this approach, when a species is identified by DNA barcoding, the tag is added to the standard DNA barcode sequence. These tags can improve the efficiency of barcoding identification by removing the overlap between intra- and interspecific genetic divergence values.

**Figure 4:**
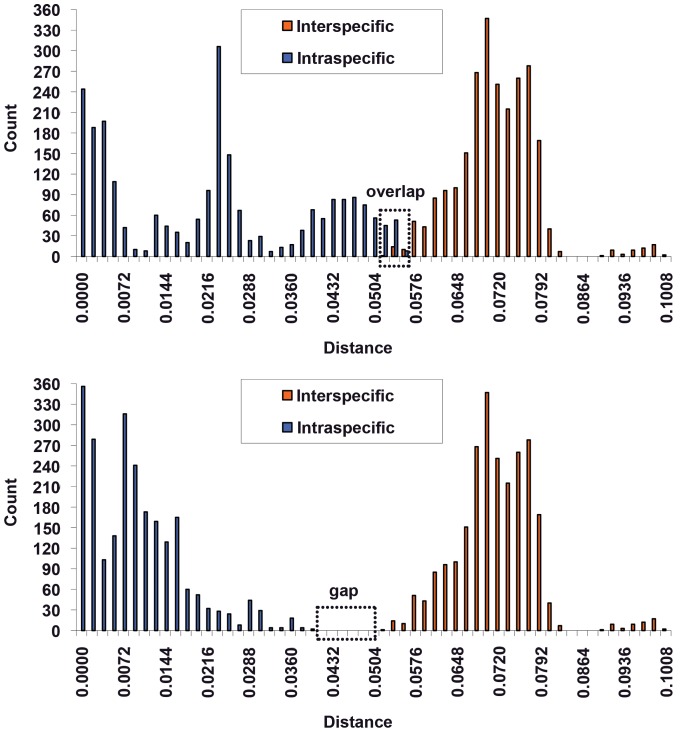
Frequency histogram of intra- and interspecific genetic divergence between *C. formosana* (Takahashi) and *C. pinea* (Zetterstedt) before (above) and after (below) using “tag barcodes.” Genetic divergence was calculated using K2P model.

This method is best illustrated using the following experimental data as an example. In traditional taxonomy, *C. formosana* (Takahashi) and *C. pinea* (Zetterstedt) are regarded as sister groups [25], and our molecular data support this view. For *C. formosana* and *C. pinea*, the mean intraspecific *COI* sequence divergence was 0.0165 and 0.0341, and the maximum distance was 0.0476 and 0.0559, respectively. A barcoding gap did not exist in these samples, with the overlapping regions ranging from 0.0506 to 0.0559 (Figure 4). Therefore, DNA barcoding would fail to identify samples that are in these regions.

“Tag barcodes” were used to resolve this problem. We first determined the composition of the tag for each species according to multiple alignments (Table 5). For example, for *C. formosana* (Takahashi), using the standard 658 bp *COI* sequence as its DNA barcode, we attached the tag (7, 49, 61, 214, 268, 340, 343, 382, 403, 427, 532) in the barcode; each number represents the standard barcode position of each highly variable site in sequence.

**Table 5:** The barcoding tags of two species, *Cinara formosana* (Takahashi) and *Cinara pinea* (Zetterstedt).

Species	Tag
*Cinara formosana* (Takahashi)	7, 49, 61, 214, 268, 340, 343, 382, 403, 427, 532
*Cinara pinea* (Zetterstedt)	7, 16, 55, 88, 94, 178, 187, 202, 223, 235, 247, 266, 301, 340, 361, 367, 385, 400, 418, 457, 468, 475, 499, 529, 533, 556, 595, 619

When we calculated the inter-specific divergence, if we retained the tag, the length of sequence is not change, so the inter-specific divergences are the same. However, if we deleted the tag when we calculated the intra-specific divergence, the length of sequences is shorter, and the intra-specific divergences are less than before. We believe that the highly variable sites have a negative impact on calculating intra-specific divergence, yet play an important role in distinguishing sequences among different species. This is due to the variable rate of evolution of certain loci in different species [26]–[28], which may interfere with the identification of individuals of the same species.

Following the application of tag barcodes, our results indicated that the average divergence between *C. formosana* and *C. pinea* was 0.0705 (ranging from 0.0506 to 0.0995) but that the average intraspecies divergence was less than previously calculated (0.0086 vs 0.0218).

A barcoding gap of 0.0393–0.0506 existed in *C. formosana* (Takahashi) and *C. pinea* (Zetterstedt). Ideally, in a DNA barcoding analysis, the intraspecies divergence and interspecies divergence should be separated by a recognizable gap [2], [29] that functions to identify members of the same species. Therefore, with the use of “tag barcodes” that reduce the divergence overlap, DNA barcoding becomes a more efficient means of species identification.

**Figure 5:**
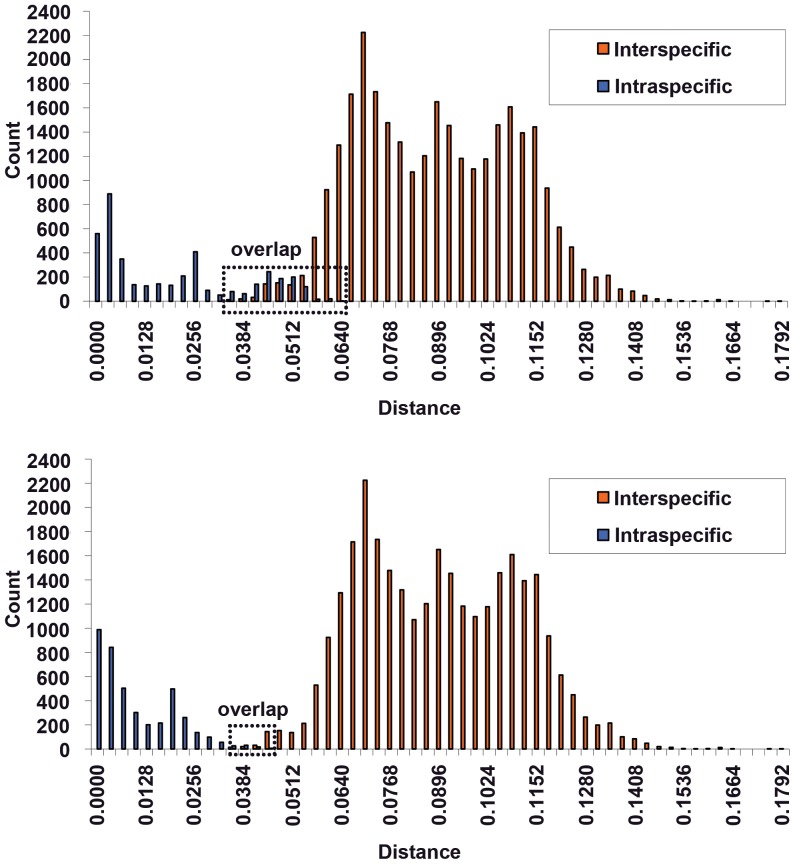
Frequency histogram of intra- and interspecific genetic divergence among Lachninae before (above) and after (below) using “tag barcodes.” Genetic divergence was calculated using K2P model.

To demonstrate the reliability of this improved method, we expanded our analysis to include a large number of Lachninae specimens. Prior to analysis, species in which the genetic divergence between congeneric species was less than 0.002 were removed; these species are likely synonymous. For the remaining species, we calculated the inter-specific and intra-specific divergence both before and after the application of tag barcodes. As a result of tag barcoding, the barcoding overlap in *COI* was nearly removed (Figure 5), and the rate of correct species identification by the “best close match” approach increased by up to 11.3% (from 83.9% to 95.2%). Similar results were observed for *COII* and *Cytb*. Therefore, “tag barcodes” can improve the accuracy of identification by introducing a gap between inter- and intraspecific divergence values.

We recommend the following approach to identify species by DNA barcoding in taxa that contain many closely related species. (i) Get the standard DNA barcoding sequences for the unknown sample. In order to ensure the accuracy of identification, more than 10 individuals for testing are better. (ii) Compare these sequences to the DNA barcoding sequences of known species and determine which species are closely related to previously unknown samples. (iii) Compare the tag among these species to find the best species match. It is also possible to calculate the intra- and interspecific genetic divergence using “tag barcodes” to find the best species match.

In summary, we suggest that specific markers suitable for different taxa should be chosen as standard DNA barcodes instead of using *COI* exclusively, which will improve the success rate of identification by DNA barcoding. By analyzing the aphid subfamily Lachninae in this study, we determined that the *COII* sequence yielded lower intra- and higher interspecific genetic divergence values for Lachninae compared to *COI* and *Cytb*. A *COII*-based identification system will therefore be more effective in identifying Lachninae species. Furthermore, the use of “tag barcodes” is a useful method through which the accuracy of identification by DNA barcoding can be improved by removing the overlap between intra- and interspecific genetic divergence. We recommended that this method be used in taxa containing many closely related species, and we suggest that this approach can be used to resolve the problem of species misidentification by conventional DNA barcoding.

## Materials and Methods

### Taxon sampling and data collection

Samples were selected to ensure the coverage of most Lachninae genera, including *Cinara* Curtis, *Eulachnus* del Guercio, *Essigella* del Guercio, *Schizolachnus* Mordvilko, *Lachnus* Burmeister, *Longistigma* Wilson, *Maculolachnus* Gaumont, *Nippolachnus* Matsumura, *Pterochloroides* Mordvilko, *Pyrolachnus* Basu *et* Hille Ris Lambers, *Tuberolachnus* Mordvilko, *Stomaphis* Walker, *Protrama* Baker and *Trama* von Heyden (Table S2). To avoid overestimating the sequence divergence between congeneric taxa due to the lack of a broad geographical sampling for a single species [4], we studied samples of selected species, including *Cinara formosana* (Takahashi), *Cinara tujafilina* (del Guercio), *Cinara pinea* (Mordvilko) and *Lachnus tropicalis* (van der Goot), which originated from geographically distant locations. The ingroup set included 1098 sequences representing 83 species, 14 genera and 3 tribes (Table S2). Among these sequences, we amplified 340 *COI* sequences, 354 *COII* sequences and 294 *Cytb* sequences and identified 110 sequences from European and North American samples that were downloaded from GenBank. The outgroups were *Pineus armandicola* (Zhang) (Adelgidae), *Phylloxerina salicis* (Lichtenstein) (Phylloxeridae), *Aphis gossypii* Glover (Aphidinae: Aphidini), *Mindarus keteleerifoliae* Zhang (Mindarinae) and *Macrosiphoniella yomogifoliae* (Shinji) (Aphidinae: Macrosiphini).

The collection information for each sample, including the location, the host plant and the collection date, is listed in Table S1. Specimens used for slide mounting were stored in 70% ethanol, while all other samples were stored in either 95% or 100% ethanol. All samples and voucher specimens were deposited in the National Zoological Museum of China, Institute of Zoology, Chinese Academy of Sciences, Beijing, China.

Each sample included several individual organisms. One to three individuals per sample were used for DNA isolation for molecular studies, and three to five individuals per sample were mounted on slides for morphological examination. Voucher specimens from all samples were identified by their main morphological diagnostic features and were compared to previously identified specimens.

### DNA extraction, PCR and sequencing

Total DNA was extracted from a single aphid preserved in 95% or 100% ethanol. Tissue homogenates were incubated at 55°C in lysis buffer (30 mM Tris-HCl [pH 8.0], 200 mM EDTA, 50 mM NaCl, 1% SDS and 100 µg/ml Proteinase K) for 5–7 hours, followed by a standard phenol-chloroform-isoamylalcohol (PCI) extraction with modifications [30]. DNA was precipitated from the supernatant with 2 volumes of cold ethanol, centrifuged, washed, dried and dissolved in 15–20 µl TE buffer and was stored at 4°C for later use.

The amplicon size of *COI* is approximately 660 bp; the primers used (5′–3′) were ATTCAACCAATCATAAAGATATTGG and TAAACTTCTGGATGTCCAAAAAATCA [10]. The amplicon size of *COII* ranges from 668 to 671 bp; the primers used (5′–3′) were CATTCATATTCAGAATTACC and GAGACCATTACTTGCTTTCAGTCATCT [31]. The amplicon size of *Cytb* ranges from 676 to 730 bp; the primers used (5′–3′) were GATGATGAAATTTTGGATC and CTAATGCAATAACTCCTCC [32]. PCR amplification of *COI* was performed with an initial denaturation of 5 min at 94°C followed by 40 cycles under the following conditions: 94°C for 30 s, 50°C for 1 min and 72°C for 1 min, with a final extension of 72°C for 10 min. PCR for *COII* was performed with an initial denaturation of 3 min at 94°C followed by 35 cycles under the following conditions: 94°C for 30 s, 46°C for 1 min and 72°C for 1 min, with a final extension of 72°C for 10 min. PCR for *Cytb* was performed with an initial denaturation of 5 min at 95°C followed by 40 cycles under the following conditions: 95°C for 1 min, 48°C for 1 min 50 s and 72°C for 1 min, with a final extension of 72°C for 10 min.

Sequencing reactions were performed bidirectionally with the appropriate amplification primers using a BigDye Terminator Cycle Sequencing Kit v.2.0 (Applied Biosystems, USA) and an ABI 3730 automated sequencer (Applied Biosystems, USA). Each of the sequence reactions was repeated three times for reproducibility.

Chromatograms of the sense and antisense sequences were assembled and analyzed using Seqman software (DNASTAR, Inc.), and a consensus sequence was obtained. Multiple alignments were generated using CLUSTALX [33] and were subsequently pruned to a length of 657 bp (*COI*), 671 bp (*COII*) or 730 bp (*Cytb*). We confirmed that these sequences were correct by translating them *in silico* using Editseq software (DNASTAR, Inc. 1996). Sequences were deposited in GenBank, and accession numbers are provided in Table S1.

### Data analysis

Nucleotide saturation was analyzed by plotting the number of transitions and transversions at each codon position against the Tamura & Nei [34] (TN93) genetic distance using DAMBE [35]. Saturation was achieved if the mutation frequency leveled off as the sequence divergence increased. Intraspecific and interspecific (between congeneric species pairs) sequence divergence was based on Kimura' 2 parameter (K2P) distances for aphid species; divergence scores were calculated by MEGA 5.0. The K2P model provided the best metric when genetic distances were low [36]. Neighbor-joining (NJ) analysis [37] was used to examine relationships among taxa and population samples.

### Identifying species based on distance

#### “Best match”

We used TaxonDNA to identify the closest barcode match for each query. If both sequences were from the same species, the identification was considered successful, whereas mismatched sequences were counted as failures. Several equally best matches from different species were considered to be an ambiguous result.

#### “Best close match”

We used TaxonDNA to plot the relative frequency of intraspecific distances to determine the threshold value below which 95% of all intraspecific distances were found. All sequences without a barcode match below this threshold value remained unidentified. For the remaining sequences, their identities were compared to the species identity of the closest barcode. If the species were identical, the identification was considered successful. The identification was considered to be a failure if the species identities were mismatched and was considered to be ambiguous if several equally best matches were found belonging to a minimum of two species.

### Attaching tags in the DNA barcode

MEGA 5.0 was used for “tag barcodes” analysis. A multiple alignment of all studied barcode sequences from one species revealed highly variable sites that we termed “tags.” The number in each tag represents the standard barcode position of each highly variable site in the sequence. Highly variable sites must vary by greater than 30% within a species. The more haplotypes are contained within a species, the more precise of tags can be found. This threshold value was derived from the statistical data obtained from Lachninae. If the threshold were lower (or too low), then there are too many tags representing a single species, and if the threshold were too high, then there may be too few tags to provide a unique identification for each species.

## Supporting Information

Figure S1 **Transitions (s, in blue) and transversions (v, in green) versus divergence for three mitochondrial gene sequences.**
(JPG)

Figure S2 **NJ analysis of K2P distances in** ***Cinara pinea*** **(Zetterstedt),** ***C. atrotibialis*** **David** ***&*** **Rajasingh and** ***C. piniphila*** **(Ratzeburg).** A, B, and C are the *COI*, *COII* and *Cytb* NJ trees, respectively.
(TIF)

Table S1 **The detailed collection information and GenBank accession numbers of Lachninae species included in this study.**
(XLS)

Table S2 **List of Lachninae species included in this study.**
(DOCX)

Table S3 **Mean intraspecific nucleotide divergence of Lachninae species generated using K2P model.**
(DOCX)
