# Correction: The Effectiveness of Three Regions in Mitochondrial Genome for Aphid DNA Barcoding: A Case in Lachininae

**DOI:** 10.1371/annotation/3595dc99-a0fc-47fc-b21b-b54003054ffd

**Published:** 2013-05-08

**Authors:** Rui Chen, Li-Yun Jiang, Ge-Xia Qiao

In the "Data analysis" section, the "Identifying species based on distance" subsection, the first sentence should read:

"We used TaxonDNA [38] to identify the closest barcode match for each query."

[38] is a new reference, available in full below:

38. Meier R, Shiyang K, Vaidya G, Ng PKL (2006) DNA barcoding and taxonomy in Diptera: a tale of high intraspecific variability and low identification success. Syst Biol 55: 715–728.

