# Supplementary figures and images for: The Effectiveness of Three Regions in Mitochondrial Genome for Aphid DNA Barcoding: A Case in Lachininae
Source: PLoS One. 2012 Oct 3;7(10):e46190. doi: 10.1371/journal.pone.0046190 (PMC3463548; doi:10.1371/journal.pone.0046190)

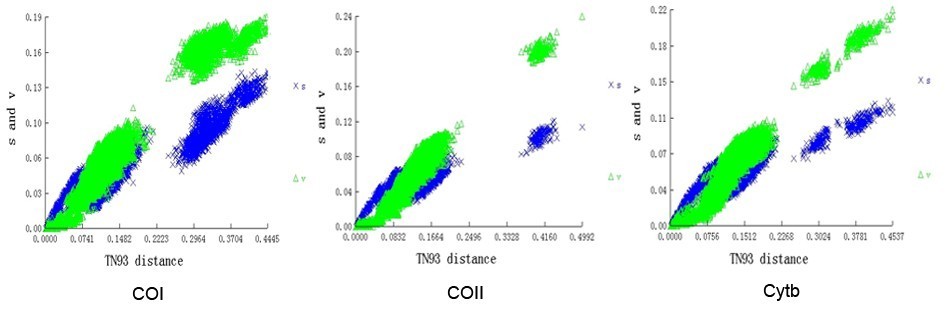

Supplement: Figure S1 — Transitions (s, in blue) and transversions (v, in green) versus divergence for three mitochondrial gene sequences. (JPG) [file pone.0046190.s001.jpg]

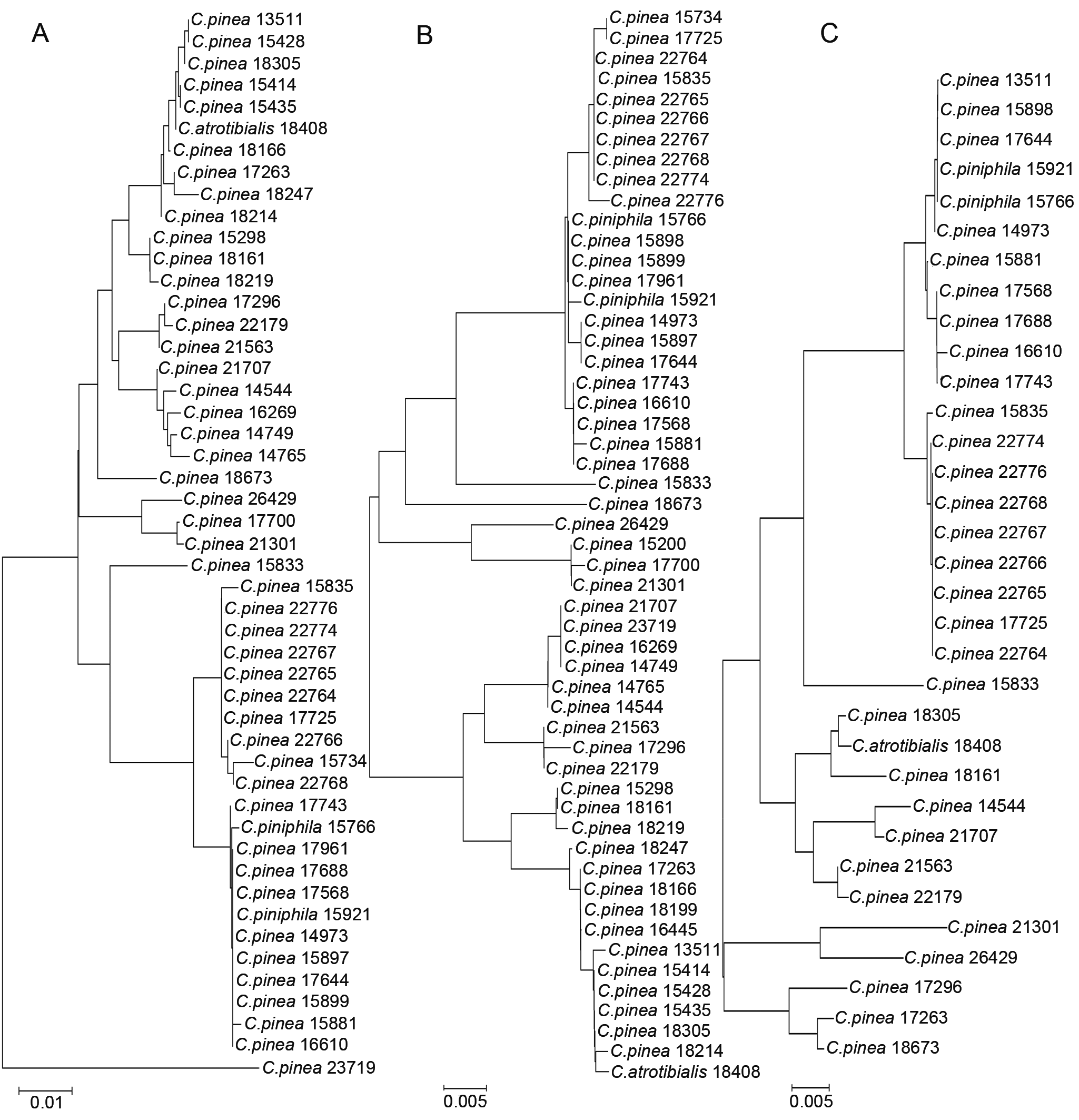

Supplement: Figure S2 — NJ analysis of K2P distances in Cinara pinea (Zetterstedt), C. atrotibialis David & Rajasingh and C. piniphila (Ratzeburg). A, B, and C are the COI, COII and Cytb NJ trees, respectively. (TIF) [file pone.0046190.s002.tif]
