# Supplementary material for: The Effectiveness of Three Regions in Mitochondrial Genome for Aphid DNA Barcoding: A Case in Lachininae
Source: PLoS One. 2012 Oct 3;7(10):e46190. doi: 10.1371/journal.pone.0046190 (PMC3463548; doi:10.1371/journal.pone.0046190)
Supplement: Table S2 — List of Lachninae species included in this study. (DOCX) [file pone.0046190.s004.docx]

Table S2. List of Lachninae species included in this study.

| Tribes | Genera | Species | No. of individuals *COI*/*COII*/*Cytb* | No. of total sequences |
| --- | --- | --- | --- | --- |
| Cinarini | *Cinara* Curtis | *Cinara formosana* (Takahashi) | 56/55/38 | 149 |
| Cinarini | *Cinara* Curtis | *Cinara atrotibialis* David *&* Rajasingh | 1/1/1 | 3 |
| Cinarini | *Cinara* Curtis | *Cinara pilicornis* (Hartig) | 19/24/19 | 62 |
| Cinarini | *Cinara* Curtis | *Cinara pinikoraiensis* Zhang | 4/4/4 | 12 |
| Cinarini | *Cinara* Curtis | *Cinara cuneomaculata* (del Guercio) | 19/19/19 | 57 |
| Cinarini | *Cinara* Curtis | *Cinara acutirostris* Hille Ris Lambers | 1/1/1 | 3 |
| Cinarini | *Cinara* Curtis | *Cinara costata* (Zetterstedt) | 6/6/5 | 17 |
| Cinarini | *Cinara* Curtis | *Cinara tujafilina* (del Guercio) | 38/32/31 | 101 |
| Cinarini | *Cinara* Curtis | *Cinara pinea* (Mordvilko) | 46/51/30 | 128 |
| Cinarini | *Cinara* Curtis | *Cinara alba* Zhang | 1/1/1 | 3 |
| Cinarini | *Cinara* Curtis | *Cinara piniarmandicola* Zhang, Zhang *&* Zhong | 19/19/17 | 55 |
| Cinarini | *Cinara* Curtis | *Cinara piniphila* (Ratzeburg) | 2/2/2 | 6 |
| Cinarini | *Cinara* Curtis | *Cinara bungeanae* Zhang, Zhang *&* Zhong | 6/6/6 | 18 |
| Cinarini | *Cinara* Curtis | *Cinara largirostris* Zhang, Zhang *&* Zhong | 9/9/9 | 27 |
| Cinarini | *Cinara* Curtis | *Cinara laricis* (Hartig) | 3/4/4 | 11 |
| Cinarini | *Cinara* Curtis | *Cinara orientalis* (Takahashi) | 1/1/1 | 3 |
| Cinarini | *Cinara* Curtis | *Cinara brevisaeta* Zhang, Zhang *&* Zhong | 2/2/2 | 6 |
| Cinarini | *Cinara* Curtis | *Cinara piceae* (Panzer) | 6/8/7 | 21 |
| Cinarini | *Cinara* Curtis | *Cinara pruiniviridis* Zhang, Chen, Zhong *&* Li | 2/2/2 | 6 |
| Cinarini | *Cinara* Curtis | *Cinara fornacula* Hottes | 4/0/0 | 4 |
| Cinarini | *Cinara* Curtis | *Cinara coloradensis* (Gillette) | 4/0/0 | 4 |
| Cinarini | *Cinara* Curtis | *Cinara shinjii* Inouye | 1/0/0 | 1 |
| Cinarini | *Cinara* Curtis | *Cinara anelia* Favret *&* Voegtlin | 1/0/0 | 1 |
| Cinarini | *Cinara* Curtis | *Cinara atlantica* (Wilson) | 2/0/0 | 2 |
| Cinarini | *Cinara* Curtis | *Cinara watsoni* Tissot | 2/0/0 | 2 |
| Cinarini | *Cinara* Curtis | *Cinara pergandei* (Wilson) | 4/0/0 | 4 |
| Cinarini | *Cinara* Curtis | *Cinara smaragdina* Pashtshenko | 1/0/0 | 1 |
| Cinarini | *Cinara* Curtis | *Cinara occidentalis* (Davidson) | 1/0/0 | 1 |
| Cinarini | *Cinara* Curtis | *Cinara longipennis* (Matsumura) | 1/0/1 | 2 |
| Cinarini | *Cinara* Curtis | *Cinara etsuhoe* Inouye | 0/1/0 | 1 |
| Cinarini | *Cinara* Curtis | *Cinara ponderosae* (Williams) | 0/1/0 | 1 |
| Cinarini | *Cinara* Curtis | *Cinara glabra* (Gillette *et* Palmer) | 0/1/0 | 1 |
| Cinarini | *Cinara* Curtis | *Cinara pseudotaxifoliae* Palmer | 0/1/0 | 1 |
| Cinarini | *Eulachnus* del Guercio | *Eulachnus pinitabulaeformis* Zhang | 2/2/2 | 6 |
| Cinarini | *Eulachnus* del Guercio | *Eulachnus nigricola* (Pašek) | 2/2/2 | 6 |
| Cinarini | *Eulachnus* del Guercio | *Eulachnus alticola* Börner | 3/4/4 | 11 |
| Cinarini | *Eulachnus* del Guercio | *Eulachnus drakontos* Zhang *&* Qiao | 4/4/4 | 12 |
| Cinarini | *Eulachnus* del Guercio | *Eulachnus rileyi* (Williams) | 2/2/2 | 6 |
| Cinarini | *Eulachnus* del Guercio | *Eulachnus piniarmandifoliae* Zhang | 1/2/2 | 5 |
| Cinarini | *Eulachnus* del Guercio | *Eulachnus brevipilosus* Börner | 0/1/0 | 1 |
| Cinarini | *Essigella* del Guercio | *Essigella fusca* Gillette *&* Palmer | 0/1/0 | 1 |
| Cinarini | *Essigella* del Guercio | *Essigella knowltoni* Hottes | 1/0/0 | 1 |
| Cinarini | *Schuzolachnus* Mordvilko | *Schizolachnus orientalis* (Takahashi) | 14/13/10 | 37 |
| Cinarini | *Schuzolachnus* Mordvilko | *Schizolachnus curvispinosus* Hottes, Essig *&* Knowlton | 1/0/0 | 1 |
| Lachnini | *Lachnus* Burmeister | *Lachnus longirostris* (Mordvilko) | 2/2/2 | 6 |
| Lachnini | *Lachnus* Burmeister | *Lachnus tropicalis* (van der Goot) | 32/31/29 | 92 |
| Lachnini | *Lachnus* Burmeister | *Lachnus siniquercus* Zhang | 5/6/6 | 17 |
| Lachnini | *Lachnus* Burmeister | *Lachnus yunlongensis* Zhang | 1/1/1 | 3 |
| Lachnini | *Lachnus* Burmeister | *Lachnus roboris* (Linnaeus) | 1/1/1 | 3 |
| Lachnini | *Lachnus* Burmeister | *Lachnus quercihabitans* (Takahashi) | 3/3/1 | 7 |
| Lachnini | *Lachnus* Burmeister | *Lachnus takahashii* Sorin | 0/1/0 | 1 |
| Lachnini | *Longistigma* Wilson | *Longistigma liquidambarus* (Takahashi) | 1/1/1 | 3 |
| Lachnini | *Longistigma* Wilson | *Longistigma caryae* (Harris) | 1/0/0 | 1 |
| Lachnini | *Maculolachnus* Gaumont | *Maculolachnus submacula* (Walker) | 3/4/3 | 10 |
| Lachnini | *Maculolachnus* Gaumont | *Maculolachnus sijpkensi* Hille Ris Lambers | 1/0/0 | 1 |
| Lachnini | *Nippolachnus* Matsumura | *Nippolachnus piri* Matsumura | 5/6/5 | 16 |
| Lachnini | *Nippolachnus* Matsumura | *Nippolachnus* sp. | 1/1/1 | 3 |
| Lachnini | *Pterochloroides* Mordvilko | *Pterochloroides persicae* (Cholodkovsky) | 19/0/9 | 28 |
| Lachnini | *Pyrolachnus* Basu *et* Hille Ris Lambers | *Pyrolachnus pyri* (Buckton) | 1/1/0 | 2 |
| Lachnini | *Pyrolachnus* Basu *et* Hille Ris Lambers | *Pyrolachnus* sp. | 1/0/0 | 1 |
| Lachnini | *Tuberolachnus* Mordvilko | *Tuberolachnus salignus* Mordvilko | 15/14/13 | 42 |
| Lachnini | *Stomaphis* Walker | *Stomaphis yanonis* Takahashi | 2/2/1 | 5 |
| Lachnini | *Stomaphis* Walker | *Stomaphis sinisalicis* Zhang *&* Zhong | 3/3/2 | 8 |
| Lachnini | *Stomaphis* Walker | *Stomaphis pistacicola* Zhang *&* Qiao | 1/1/1 | 3 |
| Lachnini | *Stomaphis* Walker | *Stomaphis quercisucta* Qiao *&* Zhang | 1/1/1 | 3 |
| Lachnini | *Stomaphis* Walker | *Stomaphis betulidahuricae* Zhang *&* Qiao | 2/2/1 | 5 |
| Lachnini | *Stomaphis* Walker | *Stomaphis quercus* (Linnaeus) | 14/1/0 | 15 |
| Lachnini | *Stomaphis* Walker | *Stomaphis aesculi* Takahashi | 0/1/0 | 1 |
| Lachnini | *Stomaphis* Walker | *Stomaphis aphananthae* Sorin | 0/1/0 | 1 |
| Lachnini | *Stomaphis* Walker | *Stomaphis fagi* Takahashi | 0/1/0 | 1 |
| Lachnini | *Stomaphis* Walker | *Stomaphis graffii* Cholodkovsky | 0/1/0 | 1 |
| Lachnini | *Stomaphis* Walker | *Stomaphis japonica* Takahashi | 0/1/0 | 1 |
| Lachnini | *Stomaphis* Walker | *Stomaphis longirostris* (Fabricius) | 0/1/0 | 1 |
| Lachnini | *Stomaphis* Walker | *Stomaphis pini* Takahashi | 0/1/0 | 1 |
| Lachnini | *Stomaphis* Walker | *Stomaphis takahashii* Sorin | 0/1/0 | 1 |
| Lachnini | *Stomaphis* Walker | *Stomaphis* sp. | 1/1/0 | 2 |
| Tramini | *Protrama* Baker | *Protrama flavescens* (Koch) | 0/1/0 | 1 |
| Tramini | *Protrama* Baker | *Protrama radicis* (Kaltenbach) | 0/1/0 | 1 |
| Tramini | *Protrama* Baker | *Protrama ranunculi* (del Guercio) | 0/1/0 | 1 |
| Tramini | *Trama* von Heyden | *Trama caudate* del Guercio | 0/1/0 | 1 |
| Tramini | *Trama* von Heyden | *Trama maritima* (Eastop) | 0/2/0 | 2 |
| Tramini | *Trama* von Heyden | *Trama rara* Mordvilko | 1/3/0 | 4 |
| Tramini | *Trama* von Heyden | *Trama troglodytes* von Heyden | 0/3/0 | 3 |
| Total | | | 409/385/304 | 1098 |
