# Supplementary material for: The Effectiveness of Three Regions in Mitochondrial Genome for Aphid DNA Barcoding: A Case in Lachininae
Source: PLoS One. 2012 Oct 3;7(10):e46190. doi: 10.1371/journal.pone.0046190 (PMC3463548; doi:10.1371/journal.pone.0046190)
Supplement: Table S3 — Mean intraspecific nucleotide divergence of Lachninae species generated using K2P model. (DOCX) [file pone.0046190.s005.docx]

Table S3. Mean intraspecific nucleotide divergence of Lachninae species generated using K2P model.

|  | Mean intraspecific nucleotide divergences | | |
| --- | --- | --- | --- |
| Species | *COI* | *COII* | *Cytb* |
| *Cinara formosana* (Takahashi) | 0.0165±0.0140 | 0.0118±0.0107 | 0.0184±0.0187 |
| *Cinara pilicornis* (Hartig) | 0.0353±0.0319 | 0.0270±0.0281 | 0.0164±0.0235 |
| *Cinara pinikoraiensis* Zhang | 0.0010±0.0008 | 0.0007±0.0008 | 0.0000±0.0000 |
| *Cinara cuneomaculata* (del Guercio) | 0.0041±0.0032 | 0.0039±0.0032 | 0.0047±0.0053 |
| *Cinara costata* (Zetterstedt) | 0.0468±0.0330 | 0.0309±0.0289 | 0.0190±0.0213 |
| *Cinara tujafilina* (del Guercio) | 0.0235±0.0234 | 0.0257±0.0266 | 0.0336±0.0325 |
| *Cinara pinea* (Mordvilko) | 0.0317±0.0177 | 0.0339±0.0196 | 0.0274±0.0186 |
| *Cinara piniarmandicola* Zhang, Zhang *&* Zhong | 0.0014±0.0013 | 0.0031±0.0025 | 0.0182±0.0160 |
| *Cinara bungeanae* Zhang, Zhang *&* Zhong | 0.0000±0.0000 | 0.0000±0.0000 | 0.0007±0.0007 |
| *Cinara largirostris* Zhang, Zhang *&* Zhong | 0.0011±0.0012 | 0.0026±0.0043 | 0.0023±0.0021 |
| *Cinara laricis* (Hartig) | 0.0171±0.0148 | 0.0197±0.0106 | 0.0293±0.0156 |
| *Cinara piceae* (Panzer) | 0.0886±0.0375 | 0.0290±0.0327 | 0.0247±0.0265 |
| *Eulachnus alticola* Börner | 0.0116±0.0075 | 0.0104±0.0063 | 0.0127±0.0074 |
| *Eulachnus drakontos* Zhang *&* Qiao | 0.0024±0.0026 | 0.0008±0.0009 | 0.0007±0.0008 |
| *Schizolachnus orientalis* (Takahashi) | 0.0295±0.0204 | 0.0396±0.0269 | 0.0502±0.0290 |
| *Lachnus tropicalis* (van der Goot) | 0.0095±0.0095 | 0.0067±0.0064 | 0.0099±0.0096 |
| *Lachnus siniquercus* Zhang | 0.0046±0.0050 | 0.0026±0.0038 | 0.0051±0.0067 |
| *Lachnus quercihabitans* (Takahashi) | 0.0112±0.0084 | 0.0071±0.0023 | / |
| *Maculolachnus submacula* (Walker) | 0.0011±0.0009 | 0.0222±0.0243 | 0.0018±0.0008 |
| *Nippolachnus piri* Matsumura | 0.0113±0.0136 | 0.0080±0.0104 | 0.0106±0.0129 |
| *Pterochloroides persicae* (Cholodkovsky) | 0.0006±0.0008 | / | 0.0047±0.0032 |
| *Tuberolachnus salignus* Mordvilko | 0.0016±0.0033 | 0.0008±0.0008 | 0.0000±0.0000 |
| *Stomaphis sinisalicis* Zhang *&* Zhong | 0.0052±0.0045 | 0.0071±0.0062 | 0.0125±0.0000 |
